# Do Shale Pore Throats Have a Threshold Diameter for Oil Storage?

**DOI:** 10.1038/srep13619

**Published:** 2015-08-28

**Authors:** Caineng Zou, Xu Jin, Rukai Zhu, Guangming Gong, Liang Sun, Jinxing Dai, Depeng Meng, Xiaoqi Wang, Jianming Li, Songtao Wu, Xiaodan Liu, Juntao Wu, Lei Jiang

**Affiliations:** 1 PetroChina Research Institute of Petroleum Exploration & Development (RIPED), Beijing, 100083, P.R. China; 2 Key Laboratory of Bio-Inspired Smart Interfacial Science, Technology of Ministry of Education, School of Chemistry and Environment, Beihang University, Beijing, 100191, P.R. China; 3 Beijing National Laboratory for Molecular Sciences (BNLMS), Institute of Chemistry, Chinese Academy of Sciences, Zhongguancun North First Street 2, Beijing, 100190, P.R. China

## Abstract

In this work, a nanoporous template with a controllable channel diameter was used to simulate the oil storage ability of shale pore throats. On the basis of the wetting behaviours at the nanoscale solid-liquid interfaces, the seepage of oil in nano-channels of different diameters was examined to accurately and systematically determine the effect of the pore diameter on the oil storage capacity. The results indicated that the lower threshold for oil storage was a pore throat of 20 nm, under certain conditions. This proposed pore size threshold provides novel, evidence-based criteria for estimating the geological reserves, recoverable reserves and economically recoverable reserves of shale oil. This new understanding of shale oil processes could revolutionize the related industries.

Shale is a new, unconventional resource; therefore, the evaluation and the exploitation of shale hydrocarbon reserves are critical to the development of related industries, the understanding of energy-based geopolitics and the building of economic infrastructures^1^,^2^,^3^. The Shale Revolution^4^,^5^,^6^ has triggered worldwide enthusiasm for research on such tight hydrocarbons, and the gains related to shale gas have been fruitful. However, similar gains have not been achieved for shale oil. In our opinion, as the birthplace of this resource^7^, shale pore throats deserve much attention because they determine the productivity of shale reservoirs^8^ and govern the storage and migration of oil and gas^9^. Additionally, a systematic and scientific understanding of shale pore throats and the occurrence state of the oil in these pore throats (continuity) is critical to assessing and estimating shale oil reserves, as well as developing corresponding recovery technologies. Although efforts^9^,^10^,^11^ have been made to determine the characteristics of liquid in shale nanopores, no specific and detailed evidence has been presented to enable the storage capacity and limitations to be elucidated. Consequently, despite the estimated high abundance of shale oil reserves^12^, shale oil reserves have not been described quantitatively.

We propose that shale oil reserves can be assessed by calculating the space in the shale pore throats (namely, the shale porosity) as long as the occurrence state of the oil in the nanopores is known. Based on the current knowledge of wetting, we consider that the occurrence state of oil is mainly determined by liquid/solid interfacial wettability, which is governed by the microscopic geometry and the interfacial chemical composition^13^,^14^,^15^,^16^,^17^. Thus, the chemical composition of shale rock and its pore sizes are keys to assessing the shale oil reserves^18^.

However, because shale pore throats are such small and complex spatial structures, further studies on shale pore throats are difficult. Taking the *Longmaxi Formation* marine shale in the Sichuan Province of China as an example, the X-ray nano-CT method was used to characterize the rock sample in 3D, showing (Fig. 1a) its porous inner structure with various pore diameters and complex components. A digital 3D spatial simulation of the pore throats (Fig. 1b) indicated that the fissured pore throats occur as long and narrow channels with a wide distribution of diameters. Statistical analysis of the pore sizes revealed that the oil storage space in shale is 90% dominated by pore throats smaller than 300 nm^19^. As the major spaces for oil storage, the pore throats of shale must be examined at the nano-scale level. How can such characterization at the nano-scale level be accomplished?

In this work, we propose to study the oil occurrence in shale pores *via* simulation of the fluidic storage and transport in a nanoporous template with a straight-channel shape and controllable diameters (see characterizations in Fig. 1c and preparations in the S.I.). Such simulations not only avoid the complexities of shale pore throats but also allow pores to be categorized into groups on the basis of the diameters of the pores, thereby enabling independent studies of the influences of pore diameters on the pore size threshold for oil storage. Such simulations offer a practical and reliable approach to scientifically evaluate oil storage and migration capacity in shale pore throats.

Although the templates can mimic the shale pores in physical shape, the templates must also exhibit wetting behaviours similar to those of shale, to fully simulate the fluidic occurrence states in shale pore throats. Using chemical vapour deposition (CVD)^20^,^21^ in the surface modification of the solids, we prepared templates with a chemical composition similar to that of shale, as illustrated in Fig. 2. The CAs and the CA variations of water and oil were tested on the surfaces of fluorinated, alkylated and untreated templates as well as on rock samples. The results indicate that oil and water have similar contact angles and identical CA variations on the surfaces of the templates and the surfaces of shale (Fig. 2). They further indicate that identification and simulation of the storage and migration of the liquid phase in shale pore throats through the use of such nanoporous templates is feasible in terms of surface adjustment. Therefore, the feasibility of using nano-channelled porous templates to simulate shale has been verified physically and chemically.

First, the templates were chemically preconditioned. Then, a staining method (see mechanism in S.I.) was used to investigate the fluidic penetration of each template under ambient conditions and under negative-pressure conditions. The results at ambient and negative pressure are presented in Table 1 and Table 2, respectively. Under ambient conditions, water and oil were unable to penetrate the 50 nm and 20 nm templates; when extra pressure was applied, the 50 nm template became penetrable. This result demonstrates that the pore throat diameter affects the fluidic penetration, i.e., a smaller space creates larger resistance. Moreover, oil and water did not penetrate the fluorinated nanopore templates unless external forces were applied, thus indicating that a lower surface free energy is a greater hindrance to liquid penetration. Alkylated templates tend to be able to allow oil to pass through water, and alkylation might facilitate the penetration of oil into the porous structures, thereby presenting a hydrophobic but oleophilic state. The results of the surface CA tests were in accord with this behaviour, as Fig. 2a,b suggested. Consequently, the templates accumulated oil and repelled water after alkylation treatment. As evident in Table 1 and Table 2, when the pore diameter was further decreased to 20 nm, neither oil nor water penetrated the material, regardless of the treatment adopted.

On the basis of the penetration tests, the following four conclusions were deduced. First, oil storage and migration in the reservoir space are determined by both the surface free energy and the size of the space; thus, lowering the surface free energy and reducing the reservoir space undermine the oil storage and migration ability of the pores. Second, the alkylated material accumulates oil and repels water. Given that the interfaces of the templates were composed of alkyl chains, making the templates similar in chemical composition to the organic-rich shale, we conclude that the organic-inorganic composite shale may contain large amounts of high quality oil with high commercial value^22^. Third, oil did not penetrate pore throats smaller than 20 nm in diameter; such pore throats constitute the lower threshold that allows the oil to flow through. Fourth, pores with diameters greater than 200 nm constitute the upper threshold: oil easily penetrated such pores regardless of the chemical or physical conditions.

The tested fluids, particularly oil, differed in their ability to penetrate the nano-channels with different diameters and compositions. According to the results of a numerical simulation (see details in S.I.), such discrepancies in liquid penetration result from differences in pore wettability. A microscopic model with a single passage was constructed to imitate the liquid wettability inside the channel with an extremely small pore throat. The results indicate that, for a channel of 50 nm, only wetting angles smaller than 60°–65° can permit free fluid outflow from the channel, completing the penetration. When the contact angle is greater than 100°–105°, the fluid remains outside. Figure 3 shows scenarios of oil flowing into nanoporous templates with pore throats of 50 nm. On these two templates, the CAs of oil droplets were 30.1° and 115.4°. Direct observations of the areas highlighted by the red frames in the inserts (Fig. 3) indicate that the numerical simulation results are valid. Moreover, Fig. 3b,c provide the most direct and visual evidence of oil penetration into a 50 nm space.

In conclusion, oil and water did not penetrate a template with pore throats smaller than the lower threshold, whereas they could flow freely into the reservoir space when the pore size was larger than the upper threshold. The lower and upper thresholds were measured and were simulated to be 20 nm and 200 nm, respectively. Because penetration is the prerequisite for fluids to be stored in the reservoir space and because the physical and chemical nature of the templates is similar to that of shale rocks, shale with pore sizes smaller than 20 nm is inferred to be of little commercial minable value, whereas shale with pore sizes greater than 200 nm has promising commercial value. In addition, geologically organic-rich resources are of higher commercial value than organic-poor resources. These proposed pore size thresholds for oil storage in shale are of revolutionary significance for estimating shale oil resources. The potential shale oil reserves estimated with the data currently available could be expanded on the basis of the lower pore size threshold. The upper pore size threshold is of theoretical significance for the estimation of economically recoverable reserves and the assessment of shale quality.

## Additional Information

**How to cite this article**: Zou, C. *et al.* Do Shale Pore Throats Have a Threshold Diameter for Oil Storage? *Sci. Rep.* **5**, 13619; doi: 10.1038/srep13619 (2015).

## Supplementary Material

Supplementary Movie 1

Supplementary Movie 2

Supplementary Information

## Figures and Tables

**Figure 1:**
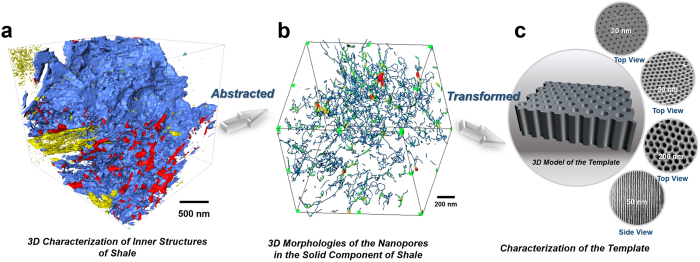
Conversion of the natural nanopores in shale into nano-channels with a regular shape and controllable diameters by using templates. (**a**) 3D inner structure image of shale captured using X-ray nano-CT and FIB-SEM (see 3D animation in S.I.). Blue denotes the rock matrix; yellow denotes the high-density mineral; and red denotes the pore system. (**b**) Specific characterization of the pores extracted from a (see 3D animation in S.I.). Statistical analysis indicates that pores smaller than 300 nm in diameter constitute greater than 90% (by vol.) of the sample. (**c**) Characterization of the templates with regular channels and controllable diameters. The studied channel diameters are 20 nm, 50 nm, 100 nm, 200 nm, 300 nm, and 500 nm. Their characterization is described in the S.I.

**Figure 2:**
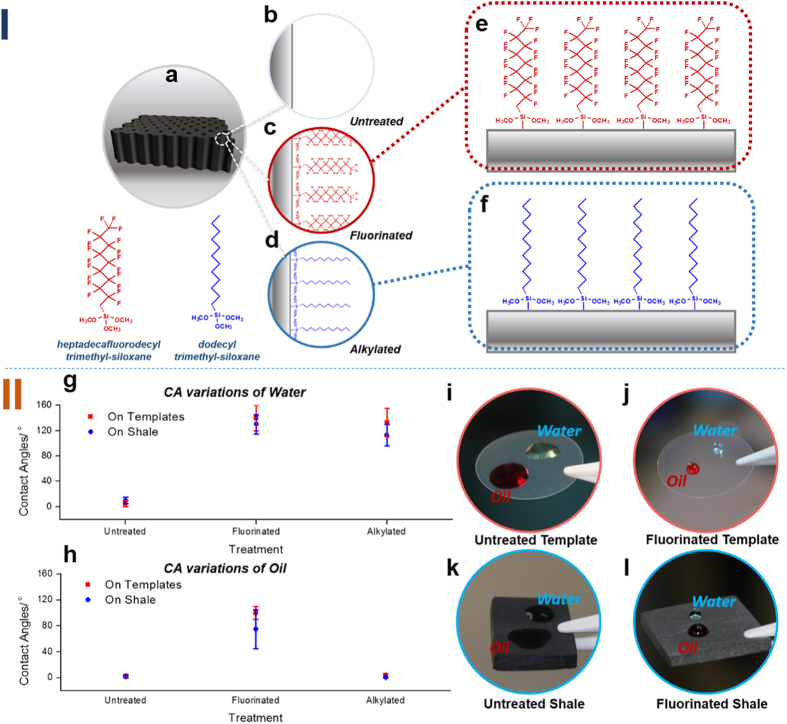
Adjustment of the surface chemical compositions of the templates to simulate the wettability of shale. **Panel I** is a schematic of the mechanism for adjusting the chemical composition *via* CVD. (**a**) General view of the structure of the templates. (**b**–**d**) Illustration of the chemical composition after the templates have been subjected to different chemical treatments. (**e**,**f**) Specific characterizations. **Panel II** shows the fluidic contact angle characterizations. (**g**) The variations of the water contact angle (CA) on templates and shale with different chemical treatments. (**h**) CA variations on the templates and shale after different chemical treatments. CA variations of water and oil exhibited similar trends on the corresponding solid surfaces. The CAs of fluids may differ on the template and shale surfaces within the same treatment because their surface micro-geometries are distinctive. (**i**–**l**) CA images of water and oil on different surfaces subjected to different treatments. The results demonstrate that simulating the wettability of shale by tuning the surface chemical composition of the templates is feasible.

**Figure 3:**
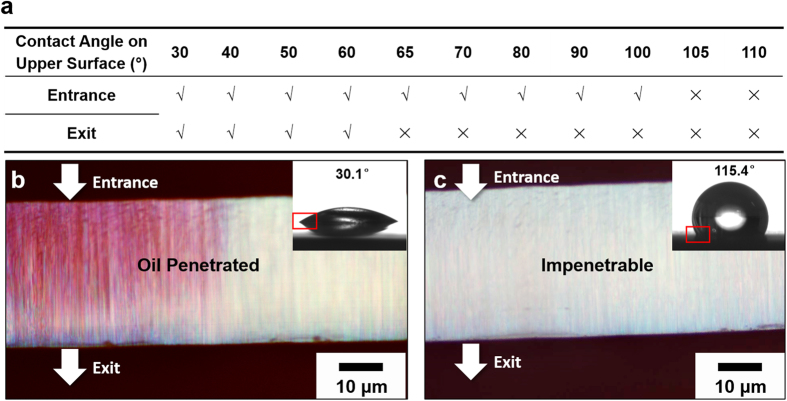
Numerical simulation and visual demonstration of oil penetrating spaces as small as 50 nm in diameter. (**a**) Numerical simulation conditions that determine the penetrability of oil liquid into a single 50 nm channel. (**b**) Optical observation demonstrating an oil droplet with a CA less than 60° on the upper, high-free-energy surface penetrated a nanoporous template with 50 nm channel diameters. (**c**) Optical observation demonstrating an oil droplet with a CA greater than 105° on a low-free-energy surface was unable to penetrate a nanoporous template with 50 nm channel diameters. (**b**,**c**) are the cross sectional view of the template observed using an optical microscope (Olympus BX51, Japan) after the Sudan-dyed oil had been removed. The insets are images of the CA of oil droplets on the corresponding templates.

**Table 1:** Fluid penetration of the templates under ambient conditions.

**Channel Diameter (nm)**	**Surficial Treatment Methods and the Tested Liquid**					
	**Untreated Water**	**Untreated Oil**	**Fluorinated Water**	**Fluorinated Oil**	**Alkylated Water**	**Alkylated Oil**
20	×	×	×	×	×	×
50	×	×	×	×	×	√
100	√	√	×	×	×	√
200	√	√	×	×	×	√
300	√	√	×	×	×	√
500	√	√	×	×	×	√

NOTES: √ Penetrable, × Impenetrable.

**Table 2:** Fluid penetration of the templates when negative pressure was applied.

**Channel Diameter (nm)**	**Surficial Treatment Methods and the Tested Liquid**					
	**Untreated Water**	**Untreated Oil**	**Fluorinated Water**	**Fluorinated Oil**	**Alkylated Water**	**Alkylated Oil**
20	×	×	×	×	×	×
50	√	√	×	×	×	√
100	√	√	×	×	×	√
200	√	√	×	×	×	√
300	√	√	×	√	×	√
500	√	√	×	√	×	√

NOTES: √ Penetrable, × Impenetrable.
