# Supplementary material for: Do Shale Pore Throats Have a Threshold Diameter for Oil Storage?
Source: Sci Rep. 2015 Aug 28;5:13619. doi: 10.1038/srep13619 (PMC4551973; doi:10.1038/srep13619)
Supplement: Supplementary Information [file srep13619-s3.doc]

SUPPORTING INFORMATION

**Do Shale Pore Throats Have a Threshold Diameter for Oil Storage?**

Caineng Zou^1^, Xu Jin^1,2^, Rukai Zhu^1^, Guangming Gong^2^, Liang Sun^1^, Jinxing Dai^1^, Depeng Meng^2^, Xiaoqi Wang^1^, Jianming Li^1^, Songtao Wu^1^, Xiaodan Liu^1^, Juntao Wu^2^ & Lei Jiang^2,3^

^1^PetroChina Research Institute of Petroleum Exploration & Devel-opment (RIPED), Beijing, 100083, P.R. China, ^2^Key Laboratory of Bio-Inspired Smart Interfacial Science, Tech-nology of Ministry of Education, School of Chemistry and Enviroment, Beihang University, Beijing, 100191, P.R. China, ^3^Beijing National Laboratory for Molecular Sciences (BNLMS), Institute of Chemistry, Chinese Academy of Sciences, Zhongguancun North First Street 2, Beijing, 100190, P.R. China.

Correspondence and requests for materials should be addressed to X. J. (jinxu@petrochina.com.cn) or J.W. (wjt@buaa.edu.cn).

1. **3D animations of the inner structure of shale and its abstracted pore channels are presented in MOVIE S1 and MOVIE S2. Characterizations of shale rocks were conducted using X-RADIA^TM^ Nano CT and FEI^TM^ FIB-SEM.**

**
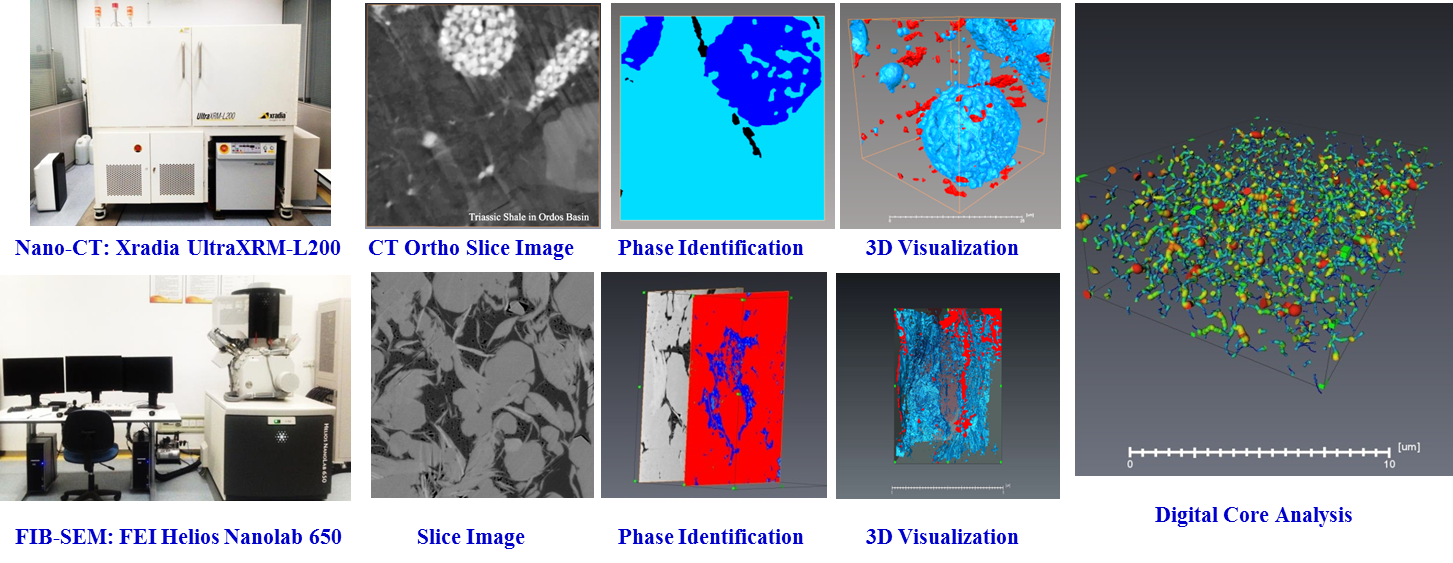
**

**Scheme S1. Characterization flow of 3D pore structure of shale rocks using Xradia UltraXRM-L200 Nano-CT and Helios 640 FIB-SEM: 1.** Slice image acquisition; 2. Phase identification and segmentation; 3. 3D reconstruction and visualization; 4. Digital core analysis and data processing.

Nano-scale X-ray Microscope (Xradia UltraXRM-L200, USA) provides insight into the microscopic structures and processes with a resolution as fine as 50 nm, which were not previously accessible with conventional lab-based X-ray technology. Producing 8 keV X-rays with excellent penetration and contrast for a wide range of samples, NanoCT enables observation of structures and materials in their natural state. The integrated phase contrast mode with the Zernike method improves the visibility of grain boundaries and material interfaces when the absorption contrast is low, enabling visibility of nano-structure.

FIB-SEM (FEI Helios Nanolab 650, USA) provides a high resolution of 1.4 nm with accelerating voltage of 1kV, and it is possible to visualize and analyze the extraordinary fine details of the pore space in both the mineral and organic matter phases of shale. The integrated focused ion beam enables us to carry out the Auto Slice & View procedure, and 3D pore structure can be extracted and visualized using Avizo Fire (An image processing software from FEI). Compared with Xradia Nano-CT, FIB-SEM has a higher 3D resolution but a rather smaller volume of interest.

The process of the 3D Characterizations of shale rock: 1. Use either Nano-CT or FIB-SEM to obtain a series of monochrome images, which both involve complex sample preparation, instrument and software setup, long time image acquisition, and post processing; 2. Use commercial software Avizo (Fire Edition) to process the images and identify different phases (e.g., organic material, minerals, pores.) based on the gray-level histogram; 3. Apply Avizo to visualize the phases of interest; 4. Use Digital Core Analysis to obtain the models for numerical simulation.

2. Preparation and characterization of the templates

**2.1 Preparation of the template:**

The ordered porous template was prepared based on the anodization process. A pure Al plate was degreased and annealed at 400 °C for 3 h in open air. The plate was then anodized under a direct current in a H_2_SO_4_ solution (0.3 M) under a constant voltage for 10 h in cryogenic surroundings. Subsequently, the pores of the anodic alumina started to form. By immersing the anodized template in a 0.36 M H_3_PO_4_ solution, an ordered porous alumina layer containing straight, parallel pores was produced. To remove the barrier layer, the anodization was continuously processed. Next, the templates were rinsed with distilled water and then dried. The pore diameters can be adjusted by tuning the voltage, the concentration of the electrolyte, the processing time, and so on.

**2.2 Characterization:**


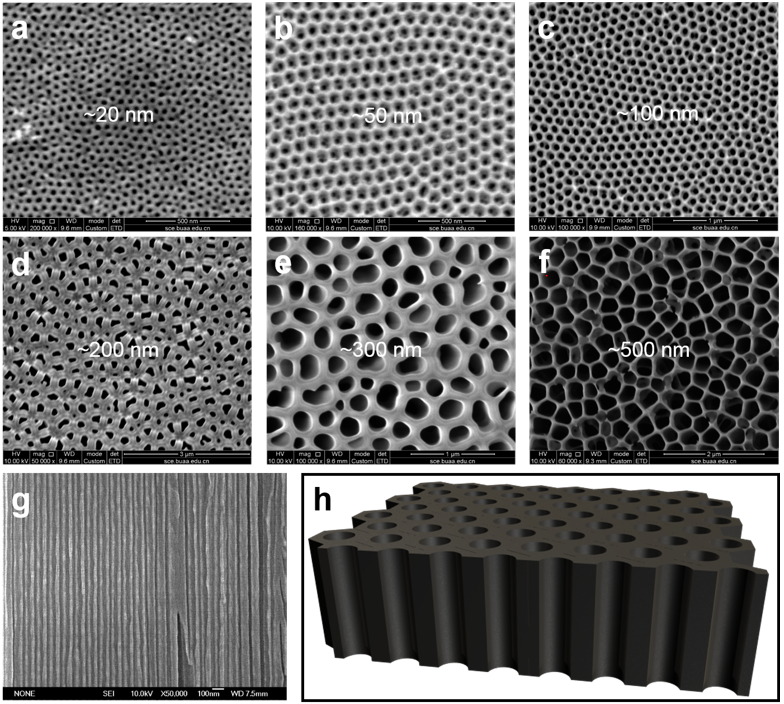


**Figure S1. SEM of the templates of different channel diameters. a-f.** Overview of the templates. **g.** Cross sectional view of the template. **h.** Graphic illustration of the 3D morphology of the template.

3. Surface treatment of the chemical composition *via* CVD


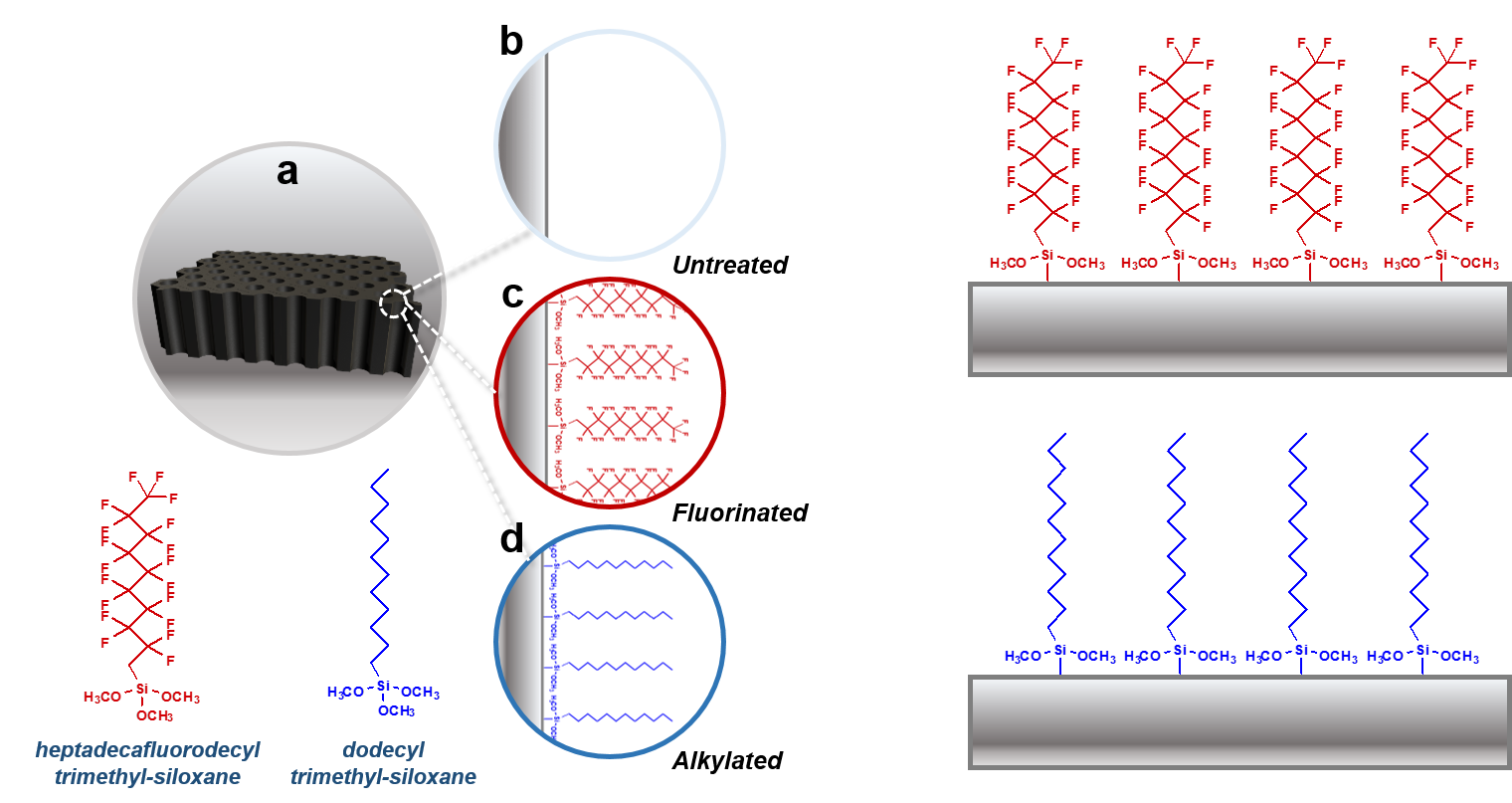


**Figure S2. Mechanism of surface treatment to change the chemical composition *via* CVD.** A liquid agent of heptadecafluorodecyl trimethyl-siloxane was added in a sealed chamber together with the templates. The chamber was evacuated to vaporize the agent, facilitating the molecules to chemically bond onto the solid’s surfaces and interfaces. The self-assembled layer replaced the original surface/interfacial area, representing a novel chemical composition of the corresponding agent on the locale. The dodecyl trimethyl-siloxane processing was similar. In our study, the heptadecafluorodecyl trimethyl-siloxane treatment was termed as fluorination and the dodecyl trimethyl-siloxane treatment was termed as alkylation. By changing the processing time and the chemical formula of the agents, different chemical compositions could be achieved.

4. Penetration determination *via* a staining method


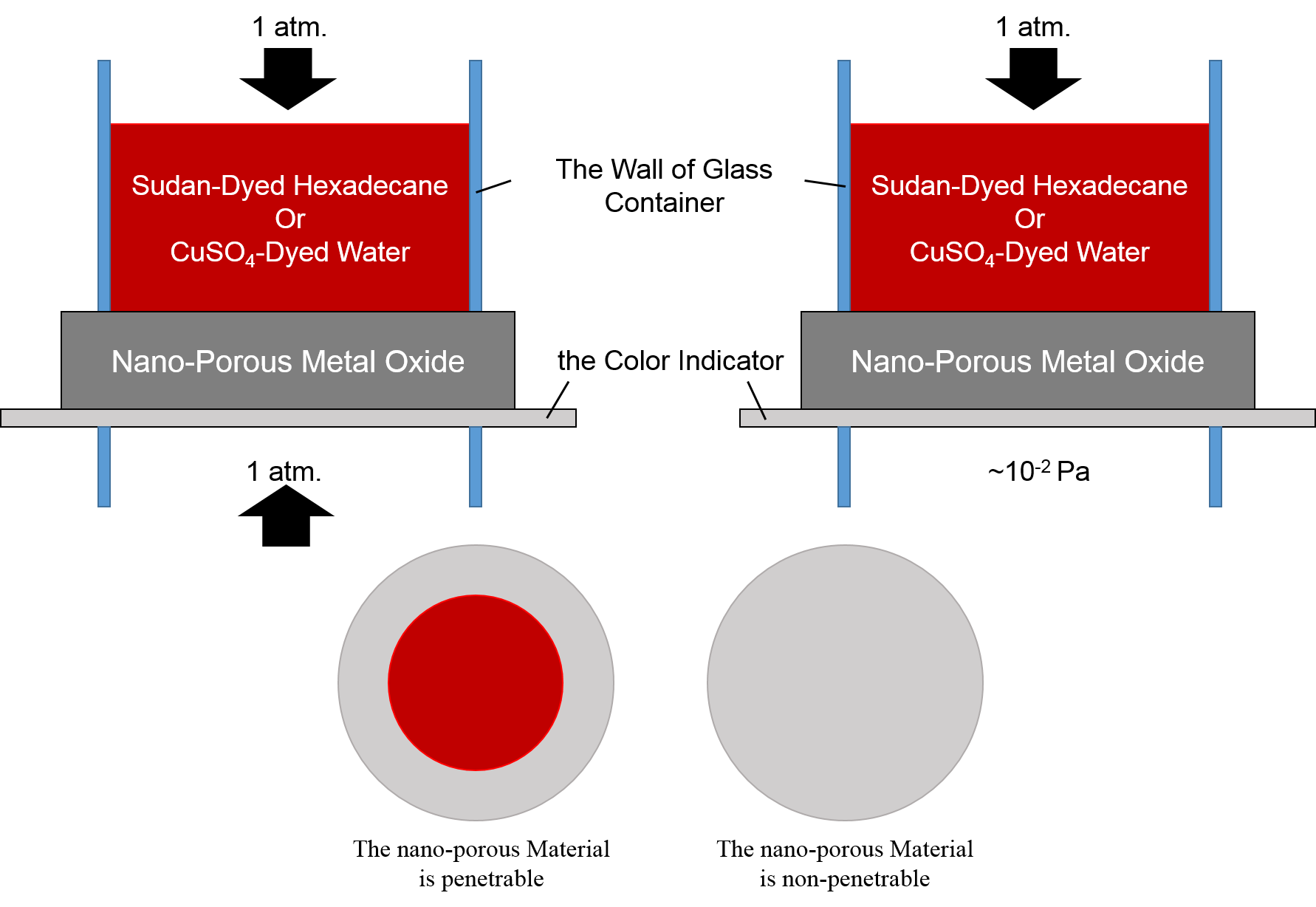


**Figure S3. Mechanism of the color mapping method to determine the fluid penetration to the templates.** Sudan-dyed oil or CuSO_4_-dyed water was added on one side of the template as shown in **a.** While in what was shown in **b.**, the air beneath the porous templet was pumped away to create driven force for the liquid. The color indicator, commonly shallow in color, was closely attached onto the other side of the template. After the proper amount of time, the indicator’s color was examined to determine whether the liquid penetrated into the template. If the indicator showed coloured stain as illustrated in **c.**, it indicated that the liquid was able to penetrate through the template. Otherwise, the unchanged indicator as shown in **d.,** demonstrated the impermeability of the template.

5. Numerical simulation of fluids with different wettability flowing through the nano-pores.


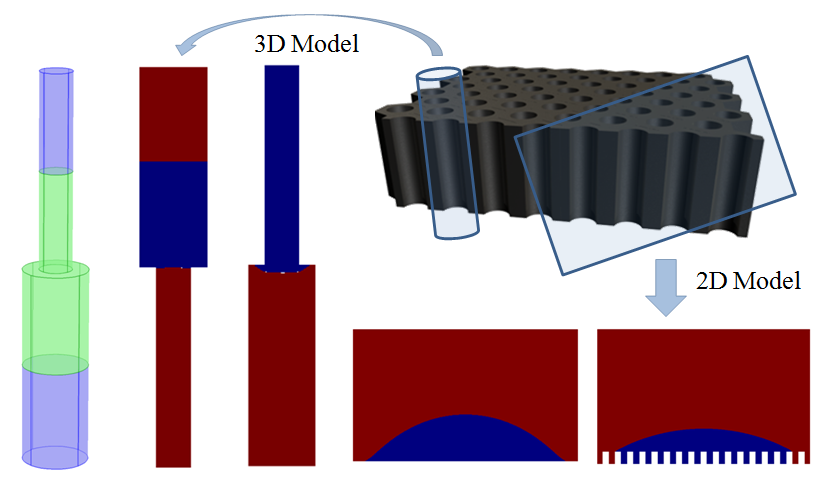


**Figure S4. Numerical simulation of fluids with different wettability flowing through the nano pores.** 3D models were established by a single nano pore with truncation of surface. 2D models were based on section cutting through a few of nano pores in together with integrated surface. Two phase flow was simulated by one fluid was pressure-injected into (or out of) the nano pore which is filled with another fuild.

The numerical simulations are based on the Incompressible Navier-Stokes Equations and Two Phase Flow. Commercial software COMSOL was used to conduct the Finite Element Analysis (FEA). The mathematical descriptions are presented below. The FEA operation can be found in the Comsol user manual (www. comsol.com).

The Navier-Stokes equations (N-S Eqs.) describe the motion of fluid substances by applying Newton's second law. These equations can describe viscous flow with the assumption that the stress in the fluid is the sum of a diffusing viscous term and a pressure term. A simplification of the N-S Eqs. can be obtained when considering an incompressible flow of a Newtonian fluid. The simplified equations include:

mass conservation equations (for incompressible flow), i.e.,

$\nabla\cdot\mathbf{u}=0$;

momentum equations, i.e.,

$\rho\frac{\partial\mathbf{u}}{\partial t}+\rho\left(\mathbf{u}\cdot\nabla\right)\mathbf{u}=\nabla\cdot\mathbf{P}+\rho\mathbf{g}+\mathbf{F}$;

constitutive law (Newtonian fluid), i.e.,

$\mathbf{P}=-p\mathbf{I}+\mu\left(\nabla\mathbf{u}+\nabla\mathbf{u}^{\mathbf{T}}\right)$;

where $\mathbf{u}$is the velocity vector ($\text{m / s}$); $\mathbf{P}$ is the stress vector ($\text{Pa}$); $\mathbf{g}$ is the gravity acceleration ($\text{m/s}^{2}$); $\rho$is the fluid density ($\text{kg/m}^{3}$); $p$ is the fluid pressure ($\text{Pa}$); $\mu$ is the dynamic viscosity ($\text{Pa}\cdot\text{s}$); $\mathbf{F}$ is the other involved forces ($\text{N}$); is the gradient operator; and $\mathbf{I}$is the unit matrix.

Two phase flow is commonly used in petroleum engineering. Two phase flow can also be described by N-S Eqs. with additional components. First, as a part of $\mathbf{F}$, the stress on the fluid surface ($\mathbf{F}_{st}$) should be considered in Equation . Next, special treatment is used to solve the two phase incompressible N-S Eqs. The commonly used phase field method (PFM) introduces several instrumental variables through the following equations (Yue et al, 2004; COMSOL, 2013):

,

,

$G=\frac{\lambda}{\varepsilon^{2}}\psi$,

where is the primary dimensionless variable; $\psi$ is the secondary dimensionless variable; $\gamma$is the fluid mobility ($\text{m}^{3}\cdot\text{s/kg}$); $f_{ext}$ is the external free energy ($\text{J/m}^{3}$); $G$is the chemical potential ($\text{J/m}^{3}$);quantity $\lambda$ is the mixing energy density ($\text{N}$), and $\varepsilon$ is a capillary width ($\text{m}$) that scales with the thickness of the interface. These latter two parameters are related to the surface tension coefficient ($\sigma$) through the equation:

$\lambda=\frac{3\varepsilon\sigma}{2\sqrt{2}}$.

The surface tension force for the phase field method is implemented as a body force, i.e.,

.

To complete the above equations, the boundary conditions should be introduced. The commonly adopted velocity boundary is defined as:

$\mathbf{u}=\pm U_{0}\mathbf{n}$,

where $\pm U_{0}$ is the normal inflow (outflow) velocity ($\text{m/s}$).

The pressure boundary condition is defined as:

$p=p_{0}$,

$\left[\mu\left(\nabla\mathbf{u}+\left(\nabla\mathbf{u}\right)^{T}\right)\right]\cdot\mathbf{n}=0$.

If wettability is considered, then the wetted wall should be introduced. For the PFM, the following equations are used for describing the wettability:

,

$\mathbf{n}\cdot\frac{\gamma\lambda}{\varepsilon^{2}}\nabla\psi=0$,

where $\theta$ is the contact angle ().
